# The Functions, Methods, and Mobility of Mitochondrial Transfer Between Cells

**DOI:** 10.3389/fonc.2021.672781

**Published:** 2021-05-10

**Authors:** Yiming Qin, Xin Jiang, Qi Yang, Jiaqi Zhao, Qiong Zhou, Yanhong Zhou

**Affiliations:** ^1^ NHC Key Laboratory of Carcinogenesis, Hunan Cancer Hospital and the Affiliated Cancer Hospital of Xiangya School of Medicine, Central South University, Changsha, China; ^2^ Cancer Research Institute, Basic School of Medicine, Central South University, Changsha, China; ^3^ Department of Neurology, Yiyang Central Hospital, Yiyang City, China

**Keywords:** mitochondria, transcellular transport, tunneling nanotubes, extracellular vesicles, Cx43 gap junction, Miro, myosin XIX

## Abstract

Mitochondria are vital organelles in cells, regulating energy metabolism and apoptosis. Mitochondrial transcellular transfer plays a crucial role during physiological and pathological conditions, such as rescuing recipient cells from bioenergetic deficit and tumorigenesis. Studies have shown several structures that conduct transcellular transfer of mitochondria, including tunneling nanotubes (TNTs), extracellular vesicles (EVs), and Cx43 gap junctions (GJs). The intra- and intercellular transfer of mitochondria is driven by a transport complex. Mitochondrial Rho small GTPase (MIRO) may be the adaptor that connects the transport complex with mitochondria, and myosin XIX is the motor protein of the transport complex, which participates in the transcellular transport of mitochondria through TNTs. In this review, the roles of TNTs, EVs, GJs, and related transport complexes in mitochondrial transcellular transfer are discussed in detail, as well as the formation mechanisms of TNTs and EVs. This review provides the basis for the development of potential clinical therapies targeting the structures of mitochondrial transcellular transfer.

## Introduction

Mitochondria are particularly important intracellular organelles, which not only play an important role in oxidative metabolism, but also have key functions in cell signaling, proliferation, metabolism, and death (1). The core functions of mitochondria in cells has led to increased research attention being paid to the transfer of mitochondria between cells, especially from the aspects of the initiation of stem cell differentiation, reprogramming of differentiated cells, and the recovery of lost mitochondrial function by receiving mitochondria from donor cells (2). Functional mitochondrial transfer between cells was first demonstrated a from mesenchymal stem cells (MSCs) to mammalian cells *via* tunneling nanotubes (TNTs) in 2006 (3). Transcellular transfer of mitochondria facilitates the incorporation of the donated mitochondria into the endogenous network of recipient cells, which results the change in the bioenergetic profile and other functional properties of recipient cell (4). This process plays an important role in diverse pathological conditions, such as repair of tissue injury, inflammatory regulation, oncogenesis and tumor drug-resistance, as well as in physical conditions maintaining tissue homeostasis (5). Studies have shown that transcellular transfer of mitochondria involves multiple mechanisms, including formation of tunneling nanotubes (TNTs), extracellular vesicles (EVs), gap junctions, exocytosis and endocytosis of naked mitochondria, cytoplasmic fusion, and other metastasis modes (6–8). In addition, transfer of mitochondria can also be used as a treatment for mitochondrial dysfunction diseases, including organ degeneration and cancer (9).

Significantly, the most common donor cells in mitochondrial transcellular transfer are stem cells, such as WJMSCs, BMMSCs and iPSC-MSCs (**Table 1**), which indicates that this process may play a crucial role in stem cell therapy (5). This type of cellular communication requires the transcellular transfer of mitochondria using all the methods mentioned above, among which tunneling nanotubes (TNTs) are the most common type of long-distance cellular connection. Mitochondria transferred by TNTs can change the metabolic and functional characteristics of the recipient cells, which has been reported in both normal cells and cancer cells, and also plays a key role in drug-resistance (41).

**Table 1:** Donor and Recipient Cells involved in the Transcellular Transport of Mitochondria.

Donor cells	Acceptor cells	Diseases/induction treatment	Mechanisms	Outcome	References
rat hippocampal astrocytes	rat hippocampal neurons and astrocytes	H_2_O_2_ or serum exhaustion	TNTs	not mentioned	(10)
Wharton’s jelly mesenchymal stem cells (WJMSCs)	rotenone-stressed MELAS fibroblasts	mitochondrial myopathy, encephalomyopathy, lactic acidosis, and stroke-like episodes (MELAS) disease	TNTs	decreased mutation and rescued mitochondrial functions	(11)
induced pluripotent stem cell-derived MSCs (iPSC-MSCs)	bronchial epithelial cells	ovalbumin- or CoCl_2_-induced mitochondrial disorder	TNTs	alleviated bronchial inflammation and apoptosis	(12)
healthy or MCA-treated human MSCs	injured hMSCs	H_2_O_2_-induced oxidative stress	TNTs	decreased oxidative stress and increased human MSC survival	(13)
BMMSCs	human umbilical vein endothelia cells	depletion of oxygen and glucose and then reoxygenation	TNTs	increased aerobic respiration, cell survival and proliferation	(14)
acute lymphoblastic leukemia cells	BMMSCs	not mentioned	TNTs	not mentioned	(15)
hMSCs	adult cardiomyocytes (CMs)	not mentioned	TNTs	metabolic reprogramming into a progenitor-like state	(16)
endothelial progenitor cells	neonatal rat CMs	not mentioned	TNTs	transformed to a cardiomyogenic phenotype	(17)
iPSC-MSCs	CMs	anthracycline-induced cardiomyopathy	TNTs	rescued injuried CMs	(18)
hMSCs	corneal epithelial cells (CECs)	rotenone-induced mitochondrial dysfunction	TNTs	alleviated oxidative stress and repaired the cornea	(19)
hMSCs	human vascular smooth muscle cells (VSMCs)	none	TNTs	promoted proliferation of MSCs	(20)
hMSCs	murine lung epithelial cells	rotenone-induced airway injury	TNTs	reduced cell death and repaired lung injury	(21)
bladder cancer cells	bladder cancer cells	spontaneously	TNTs	invasiveness of cancer cells increased	(22)
cancer-associated fibroblasts (CAFs) with high glycolysis	prostate cancer cells	none	TNTs	increased aerobic respiratory and the level of OXPHOS ATP	(23)
human lung-derived mesenchymal stromal cells	lung epithelial cells	not mentioned	TNTs EVs	repaired the damage of bronchial epithelial cells	(24)
astrocytes	neurons	ischemic damage	EVs	increased ATP and cell survival	(25, 26)
neurons	astrocytes	not mentioned	EVs	not mentioned	(26)
retinal ganglion cells	adjacent astrocytes	rotenone treatment	EVs	mitochondrial mitophagy to transcellular degradation	(27)
normal human astrocytes (HA)	human glioma cells (U87)	serum starvation	EVs	increased aerobic respiration and radiosensitivity	(28)
BMMSCs	macrophage, lung alveolar epithelial and endothelial cells	not mentioned	EVs	increased mitochondrial ROS and down-regulated TLR signaling proinflammatory cytokines	(29)
BMMSCs	macrophages	LPS-induced acute respiratory distress syndrome (ARDS)	EVs	transformed macrophagy to the anti-inflammatory and highly phagocytic phenotype and alleviated lung injury	(30)
BMMSCs	macrophages	oxidative stress	EVs	promoted bioenergy of macrophage	(31)
rat cortical astrocytes	rat neurons	not mentioned	EVs	increased ATP level and cell survival	(32)
airway myeloid-derived regulatory cells (MDRCs)	peripheral T cells	not mentioned	EVs	generated ROS and involved the bioenergetic and redox regulation	(33)
bone marrow-derived MS-5 cell line	AML cells	cytarabine	endocytosis	increased ATP level and AML cells survival from chemotherapy	(34)
bone marrow mesenchymal stromal cells (BMMSCs)	mouse alveolar epithelial cells	LPS-induced lung injury	endocytosis TNTs Cx43-GJs EVs	increased ATP, secretion of pulmonary surfactant, and survival of pulmonary cells	(35)
MSCs	bronchial epithelial cells	not mentioned	endocytosis TNTs Cx43-GJs	not mentioned	(36)
WJMSCs	mitochondrial DNA (mtDNA)-depleted ρ (0) cells	none	not mentioned	up-regulated the expression of mtDNA-encoded proteins; increased O_2_ consumption and aerobic respiratory; acquired attachment-free proliferation, cell survival and motility	(37)
MSCs	peripheral blood mononuclear cells (PBMCs) and CD3+ T cells	none	not mentioned	improved T cell activation and T-regulatory (Treg) cell differentiation; alleviated inflammatory reaction	(38)
PBMCs	UVR-damaged cells	ultraviolet radiation (UVR) damage	not mentioned	repaired UVR damage and increased viability	(39)
MSCs	human ovarian and breast cancer cell	doxorubicin	not mentioned	acquired drug-resistance to doxorubicin	(40)

Mitochondria are highly dynamic organelles with constant shape and positional change, which allows the quality control of mitochondria and mitochondrial transport to the region where ATP is needed (42). This movement is based on the cytoskeleton. Generally, the microtubule cytoskeleton is the most common way of intracellular movement, while intercellular movement is based on two forms of the microfilament cytoskeleton, extracellular vesicles (EVs) and TNTs (43). Meanwhile, the transport complex, which anchors mitochondria to the cytoskeletons, provides the power that drives mitochondrial movement.

## Physiological Function of the Transcellular Transfer of Mitochondria

Intercellular communication is important for the maintenance of physiological functions and the development pathological processes. Intercellular communication can be divided into: Between cells that are far apart, for which signal molecules, such as cytokines or hormones, can be secreted to bind to specific receptors on target cells to transmit regulatory signals and activate specific cellular activities; and gap junctions (GJs) or synapses used between adjacent cells to transfer small molecules, such as inorganic ions, neurotransmitters, reactive oxygen species (ROS) or small molecular proteins, which allows the exchange of electrical or chemical signals between these two cells (44, 45). In recent years, many new communication forms have been discovered, such as TNTs and EVs, which structures are capable of intercellular exchange of ions, small molecules such as ATP, and certain organelles [like lysosomes and autophagosomes (46)].

**Figure 1:**
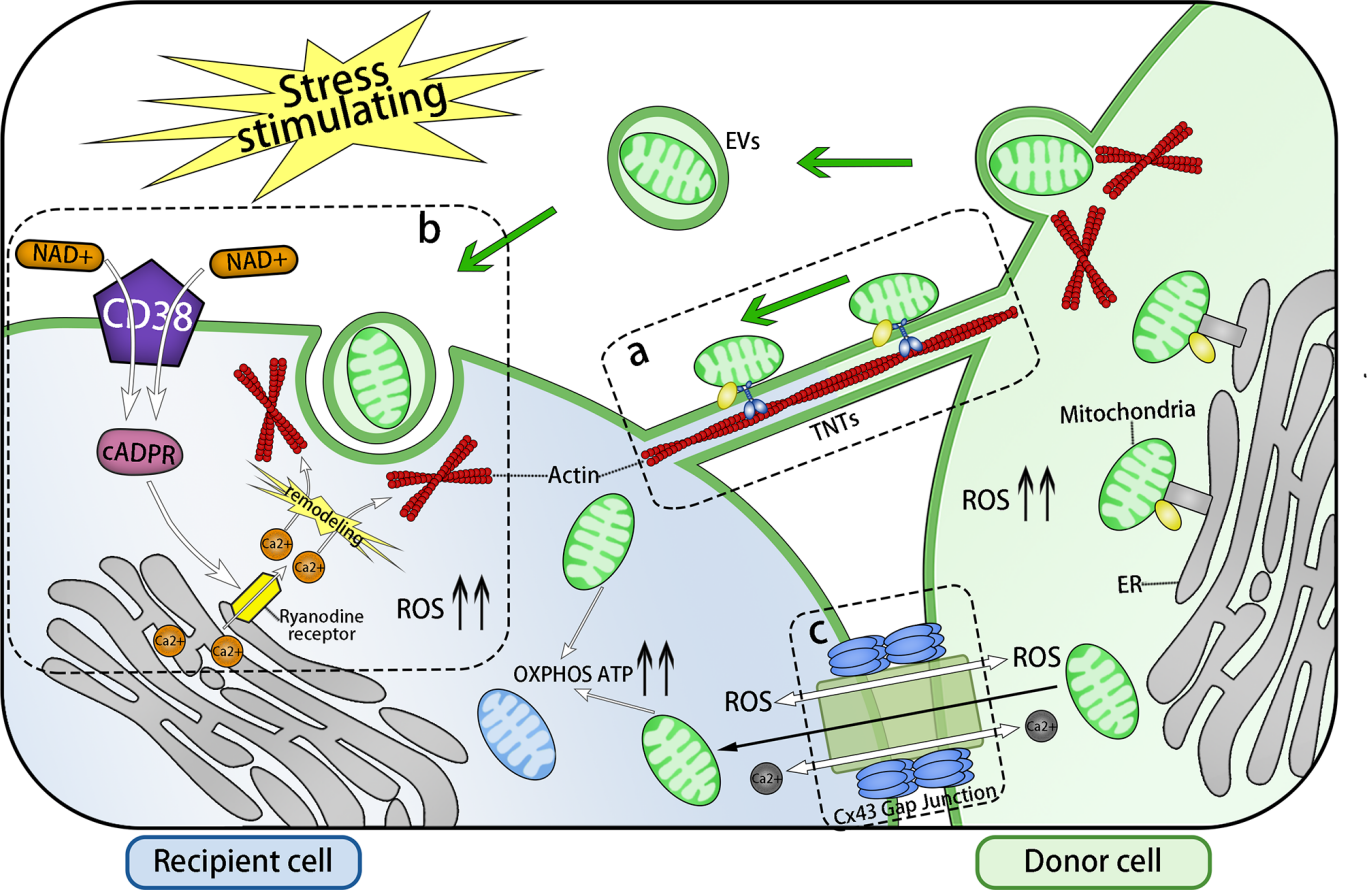
Three Main Forms of Intercellular Communication Related to Transcellular Transfer of Mitochondria. Under the stimulation of energy stress, inflammatory stimulation, or DNA damage, intracellular ROS levels increase and three forms of intercellular communication related to mitochondrial transcellular transfer are formed: TNTs, EVs, and GJs. **(A)** TNTs connects the cytoplasm of two cells, whose main framework is F-actin. Mitochondria are anchored to the actin skeleton by specific transport complex and driven by them to move from one cell to another *via* TNTS. **(B)** EV endocytosis is mediated by the NAD+/CD38/cADPR/Ca2+ pathway: Intracellular NAD+ increases and transfers to the extracellular environment under stress conditions. Then, NAD+ is catalyzed by activated transmembrane protein CD38 to generate cADPR, a second messenger controlling the release of the intracellular Ca^2+^ pool. cADPR acts on Ryanodine receptors (RyRs) on the endoplasmic reticulum, which leads to an increase in the intracellular Ca^2+^ concentration, following the remodeling of the actin cytoskeleton and invagination of cell membrane, thereby completing the endocytosis of EVs. The release of EVs and the formation of TNTs might also be mediated by this pathway. Through transcellular transfer of mitochondria, based on the actin cytoskeleton, the OXPHOS, the ATP level, and the viability of the recipient cells are all improved. **(C)** Cx43 gap junction channel (GJCs) also take part in the transcellular transfer of mitochondria. There are three possible pathways: Ca2+ or ROS exchange *via* Cx43 GJCs to modulate the formation of channels transferring mitochondria, or the direct transfer of mitochondria. ERMES: a complex that anchors mitochondria to endoplasmic reticulum.

Mitochondria are vital cellular organelles, regulating energy metabolism and cell apoptosis, which are directly related to cell survival (47). Recent studies have shown that mitochondria can also undergo intercellular transfer. The transcellular transfer of mitochondria plays different roles in different situations:

A. the quality control of mitochondria. When damage or senescence appears in mitochondria, cells can deal with injured mitochondria through mitochondrial fusion, division, mitophagy or transmitophagy; meanwhile, healthy mitochondria can be received from surrounding cells through TNTs or EVs (42), or damaged mitochondria can be transferred into surrounding cells for degradation (27, 48), thus achieving complete quality control of mitochondria, which aims to maintain the population and physiological function of mitochondria (42).
B. the rescue of cells from oxidative stress. When cells suffer oxidative stress (such as cell ischemia/hypoxia, DNA damage, and inflammatory factor stimulation), specific signals of stress, such as ROS, are activated, leading to the formation of TNTs connecting to adjacent cells or the secretion of EVs, resulting in the transfer of healthy mitochondria to stressed cells, which will correct aerobic respiration and improve the level of oxidative phosphorylation (OXPHOS) and ATP of the stressed cells rapidly (**Figure 1**) (41).

Transcellular transfer of mitochondria is a very common phenomenon in pathological conditions. Bone marrow stromal cells (BMSCs) can transport functional mitochondria to alveolar epithelial cells to counter acute lung injury caused by endotoxins (49). Under the condition of ischemia or hypoxia, astrocytes release mitochondrial granules, which are then are absorbed by neurons *via* endocytosis to improve the viability of neurons (25, 28). MSCs can infuse mitochondria into a variety of cells, such as vascular smooth muscle cells (20), pulmonary epithelial cells (21, 50), myocardial cells (51), and tumor cells, for example in ovarian cancer and breast cancer (40, 52), which is of great significance for wound healing, immune regulation, maintenance of tissue homeostasis and tumor proliferation (53). Mitochondrial transfer also occurs among monocyte derived macrophages (54). Similarly, tumor cells can induce the transcellular transfer of mitochondria. In multiple myeloma, MSCs deliver mitochondria to myeloma cells through TNTs, promoting their proliferation (55). For pulmonary adenocarcinoma A549 cells, which lose mitochondria during drug treatment, their metabolism can be restored after receiving healthy mitochondria delivered from other cells, making them more invasive (3). In general, transfer of mitochondria can ameliorate aerobic metabolism, restore energy support, and postpone apoptosis of the recipient cells, which acts a protective role in ischemic diseases or other cell dysfunctions; meanwhile, in malignant tumors, it can lead to tumor proliferation and drug resistance. Interestingly, another study seemed to propose the opposite. Normal astrocytes deliver mitochondria to glioma cells, which inhibits their proliferation. In this process, the expression levels of genes related to the tricarboxylic acid cycle were upregulated; aerobic respiration was enhanced, while glycolysis was weakened; and the mitochondrial apoptotic pathway was activated. Meanwhile, the sensitivity of tumors to radiotherapy was increased (28). These changes might be related to the inhibition of the Warburg effect in tumor cells. The following table summarizes some cases of mitochondria transfer between diverse donor and recipient cells (**Table 1**).

Furthermore, there was also spontaneous transcellular transfer of mitochondria under physical conditions. This phenomenon has been detected under conditions without stimulating factors between mouse cardiomyocytes (CMs) and human multipotent adipose-derived stem cells (hMADs) (16), renal tubular cells (RTCs) and mesenchymal multipotent stromal cells (MMSCs) (56), human vascular smooth muscle cells (hVSMCs) and BMMSCs (20), etc. It plays a potential crucial role in tissue homeostasis and needs further study.

## Methods of Transcellular Transfer of Mitochondria

### Transfer *via* TNTs

**Figure 2:**
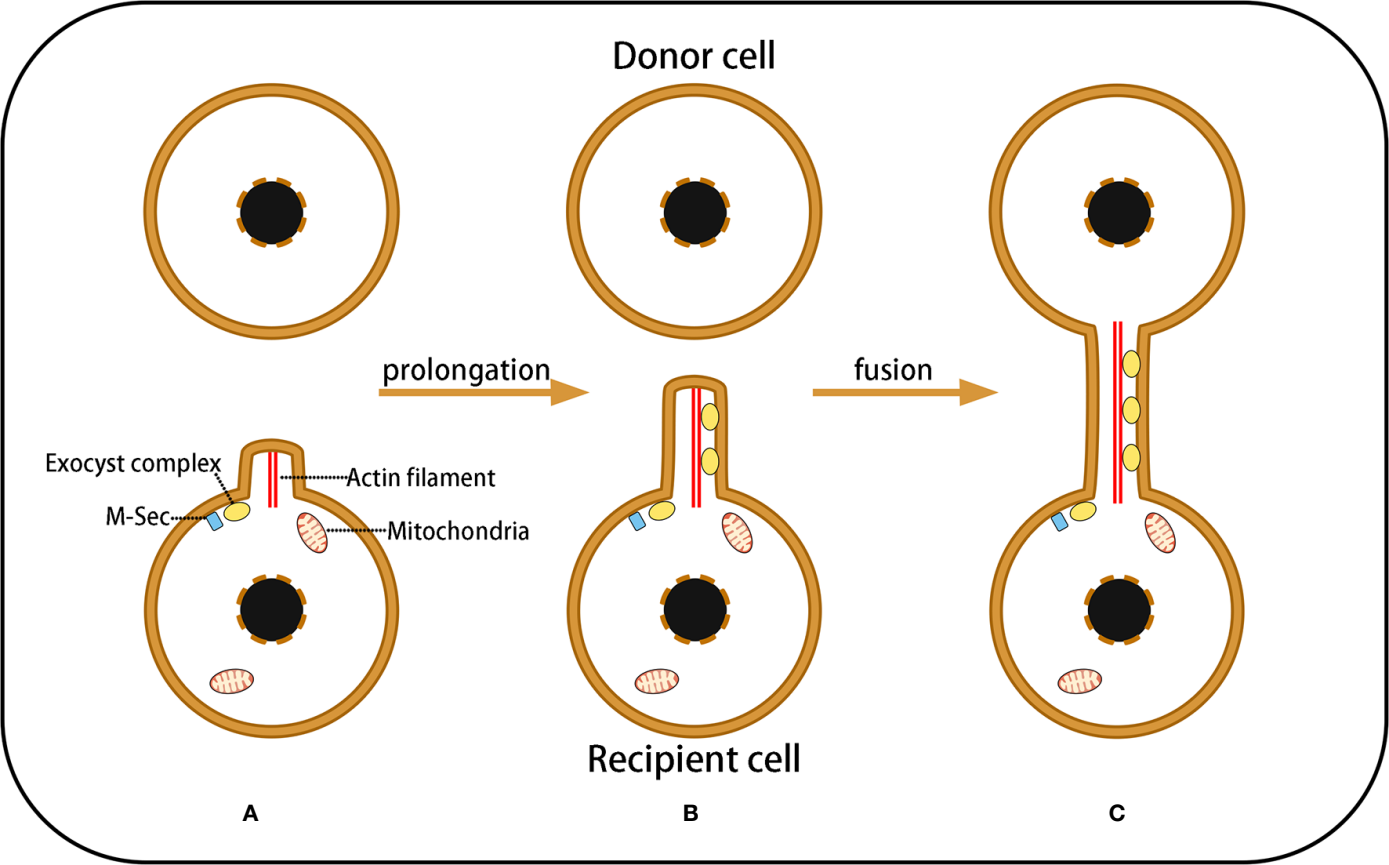
Three Stages of TNT Formation. **(A)** Under the control of M-sec and exocyst complexes, cells needing healthy mitochondria emit a membrane protrusion containing F-actin; **(B)** Exocyst complexes induce F-actin remodeling, resulting in membrane protrusion prolongation that forms a filopodium-like membrane structure; **(C)** The prolongated membrane protrusion contacts the target cell (mitochondrial donor cell) and fuses with the target cell membrane to form a membrane channel connecting the cytoplasm of the two cells, which is termed a TNs. However, the fusion mechanism of the phospholipid bilayers of the donor and recipient cells remains unclear.

Tunneling nanotubes (TNTs) are a type of long membrane structure of 100–800 nm in width and 100 μm in length. Based on F-actin as their framework, TNTs are wrapped by a phospholipid bilayer extending from the cell membrane, connecting the cytoplasm of two cells (57). TNTs allow bidirectional and unidirectional substance transport, including a variety of small molecules, proteins, organelles, and even virus particles (58–60). The formation of TNTs has three stages: Formation of a membrane protrusion, elongation of the membrane protrusion, and fusion of the membrane protrusion with the target cell membrane. Transformation of the membrane protrusion is the direct result of actin cytoskeleton remodeling (**Figure 2**). TNTs exist extensively in a variety of physiological and pathological cells, such as kidney cells (61), PC12 pheochromocytoma cells (62), astrocytes (10), and myocardial cells (17, 63).

Studies have demonstrated that TNTs act as a channel for mitochondrial transfer between various cells (**Figure 1A**; **Table 1**). Under condition of oxidative stress, intracellular p53 expression is upregulated and the AKT-PI3K-mTOR signaling pathway is activated, leading to the formation of TNTs from stressed cells to non-stressed cells, and mediating the transcellular transport of four organelles (ER, Golgi, endosome and mitochondria), including mitochondria (10). Interestingly, an experiment conducted two years later contradicted this view, concluding that TNTs did not depend on p53 activation (64). In the bone marrow microenvironment of multiple myeloma, TNT-mediated transcellular transfer of mitochondria was found to rely on the expression of CD38 (55). Although the upstream activation signals may be distinct, there should be a common downstream mechanism guiding the formation of TNTs.

A variety of molecules are involved in the process of TNT formation, including M−Sec (also known as TNF alpha induced protein 2), the exocyst complex, small GTPases, and leukocyte specific transcript 1 (LST1). The exocyst complex, an octameric protein complex, mediates the fusion of secretory vesicles derived from the Golgi body with the plasma membrane during exocytosis of yeast, mammals, and other eukaryotes (65–68). In recent years, it has been found that the exocyst complex also plays a significant role in various processes involved in the morphological transformation of the cell membrane, such as neurites, cilia, filopodia, and EVs (67, 69, 70). The components of the exocyst complex interact with several small GTPases (Rho1, Rho3, Cdc42, and RalA) to promote actin cytoskeletal remodeling, which is closely related to the function of the exocyst complex (69, 71, 72). The exocyst complex also participates in the formation of TNTs, and this process is controlled by M-Sec, a protein that has been proven to control the formation of TNTs (73, 74).

M-Sec can promote the assembly of the exocyst complex and interact with it, as well as with RalA (or Cdc42), leading to actin cytoskeleton remodeling, which is the key step of TNT formation (75). Studies have shown that in the initial stage of TNT formation, the N-terminal polybasic region of M-Sec directly integrates with phosphatidylinositol (4, 5)-diphosphate, so that M-Sec is fixed on the cell membrane. In addition, the interaction between M-Sec and RalA requires a positively charged surface at the C-terminal of M-Sec (76). Filopodia transformation is also a possible mechanism for TNT formation (62), as Cdc42, whose activated protein Ral binding protein 1 (RalBP1) regulates actin remodeling during filopodia formation (77), also participates in TNT formation; however, Cdc42 seems to be only related to the prolongation stage of TNTs (75).

**Figure 3:**
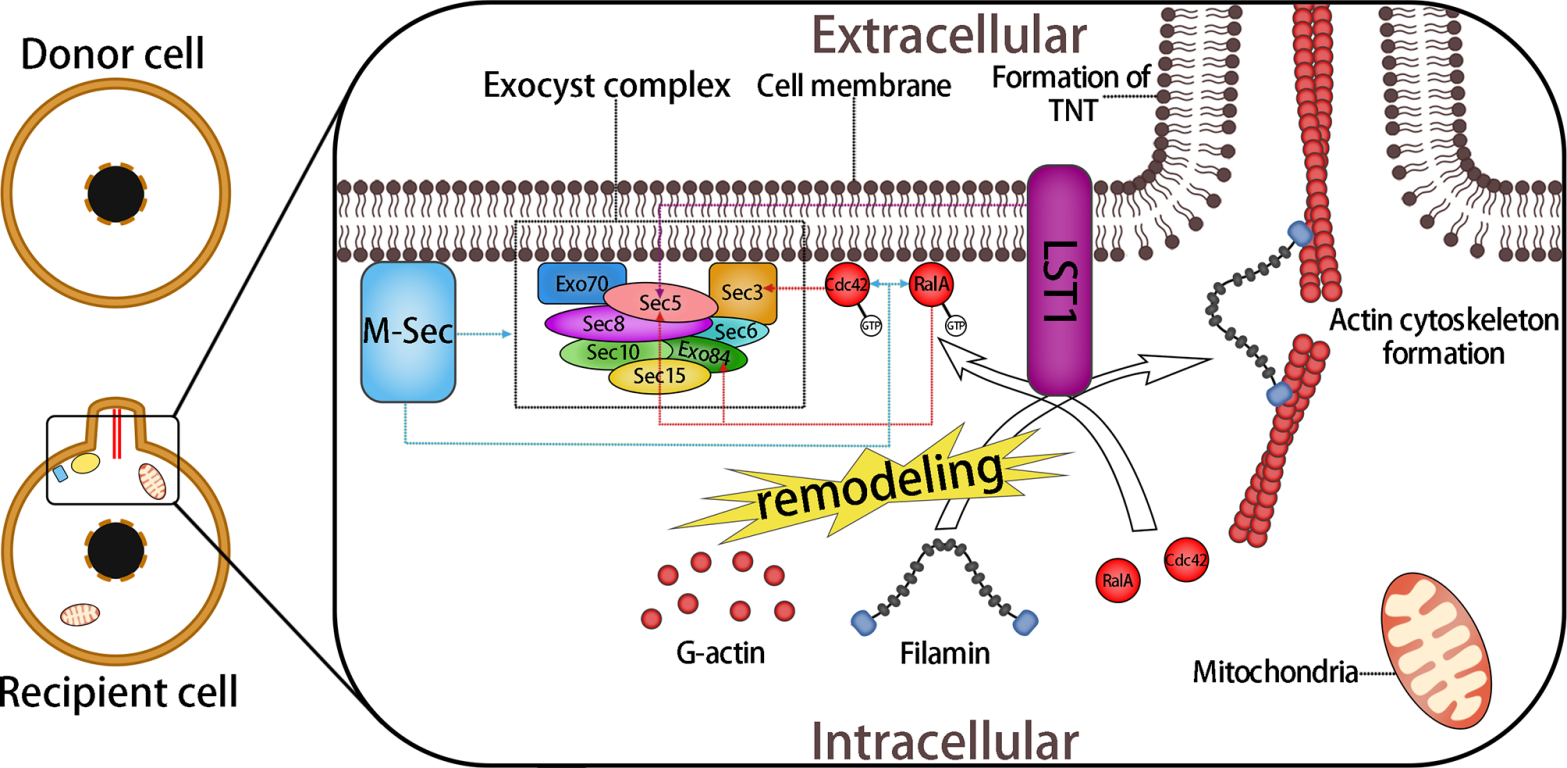
Formation of TNTs Mediated by M-Sec and the Exocyst Complex. The exocyst complex consists of eight proteins: Sec3, Sec35, Sec36, Sec38, Sec310, Sec315, Exo70, and Exo84. During the interactions of M-sec with the exocyst complex, small GTPase RalA and Cdc42 promote the assembly of the exocyst complex and lead to the remodeling of actin. Active RalA (RalA-GTP) interacts with two components of the exocyst complex, Sec5 and Exo84, which bind to RalA competitively. This interaction leads directly to the aggregation of G-actin and the subsequent formation of the membrane protrusion. The combination of Cdc42 and Sec3 plays a role in the prolongation stage of the membrane protrusion. In addition, LST1 recruits RalA to the submembrane region, and promotes its interaction with the exocyst complex and recruits filamin, an actin cross-linked protein. Moreover, LST1 can interact with M-sec, myosin, and myoferlin, which might also participate in the subsequent process of mitochondrial anchoring and transfer. Overall, a variety of molecules are involved in the formation of the membrane protrusion, including some of the molecules mentioned later. Together, they form a multi-molecular complex that regulates the formation of TNTs. Dotted arrows indicate interactions between proteins.

The MHC class III protein, LST1, is a transmembrane protein that can recruit RalA to the submembrane region and accelerates interaction between RalA and the exocyst complex. Meanwhile, LST1 recruits the actin cross-linked protein filamin. LST1, along with M-Sec, RalA, and the exocyst complex, together promote the remodeling and cross-linking of actin filaments, leading to cell membrane protrusion and fusion, ultimately leading to TNT formation (78) (**Figure 3**). However, the mechanism of the fusion between the membrane protrusion and the target cells remains unclear.

The transport and recycling of vesicles between biomembranes is also thought to be related to TNTs, as both the small GTPase Rab11a, which controls this process, and the downstream Rab8a, are involved in the formation of TNTs (79). In bladder cancer cells, inhibition of RalGPS2, the guanine nucleotide exchange factor (GEF) of RalA, also leads to a large reduction in TNT formation; moreover, RalGPS2 also interacts with LST1 and RalA and participates in the formation of multi-molecular complexes (80). In neurons, the unconventional molecular motor myosin X is also necessary for the formation of TNTs, because TNTs can be developed from filopodia driven by myosin X (81). The concentration gradient of S100 calcium binding protein A (S100A) in nervous tissue determines the extension direction of TNTs. Under oxidative stress, a decrease in S100A levels in neurons leads to a concentration gradient of S100A between astrocytes and neurons. TNTs then extend from neurons with a low concentration S100A and are received by astrocytes with a high concentration S100A (82).

TNT-mediated mitochondrial transfer can act as a survival pathway for cells under stress, for example, saving ischemic damaged cells (83), protecting the alveolar epithelium from injury (49), and repairing tissue (2, 6, 84). TNTs also exist in cancer cells, in which they are related to the survival and drug resistance of cancer cells (41, 85).

### Transfer *via* Extracellular Vesicles

There are two types of EVs: Exosomes (30–150 nm in diameter) and microvesicles (30–1000 nm in diameter) (86). As a form of intercellular communication, EVs can deliver various substances, including small molecules, organelles, and membrane proteins, and thus play a role in mitochondrial transcellular transfer in certain types of cells.

Under conditions of evacuating serum *in vitro* and stimulating ischemic stroke *in vivo*, astrocytes were able to release EVs containing mitochondria for phagocytosis and utilization by nearby neurons to reduce nerve damage (25). In a model of allergic airway disease, EVs containing mitochondria derived from airway myeloid-derived regulatory cells (MDRCs) could be ingested by peripheral T cells, resulting in modulation of the bioenergetics of T cells, which is related to immunomodulation and the regulation of inflammatory responses of the disease (33, 87). Retinal ganglion cells can produce cell membrane processes containing axon mitochondria and release vesicles, which are phagocytized and degraded by astrocytes in the optic nerve papilla (27). EVs containing mitochondria were also found in the plasma of patients with melanoma (88).

The NAD+/CD38/cADPR/Ca^2+^ pathway is the key in EV-mediated mitochondrial transcellular transfer. CD38, a transmembrane protein expressed on various cell membranes, can produce cyclic ADP-ribose (cADPR) using co-enzyme nicotinamide adenine dinucleotide (NAD) as a substrate. cADPR is a second messenger that can open the intracellular Ca^2+^ pool. cADPR acts on ryanodine receptors (RyRs) (a calcium ion channel) on the endoplasmic reticulum, which promotes the release of Ca^2+^ stored in the endoplasmic reticulum. Thus, the transient Ca^2+^ concentration in the cytosol increases, leading to a series of downstream responses (89).

Glioma cells under starvation can accumulate a large amount of NAD+ and release it into the extracellular region. Through the NAD+/CD38/cADPR/Ca^2+^ pathway, the increasing calcium in the cytosol promotes actin cytoskeleton remodeling, leading to cell membrane invagination, thus completing the endocytosis of EVs (28) (**Figure 1B**). Inhibition of endocytosis resulted in a reduction of mitochondrial transfer from bone marrow mesenchymal stem cells (BMMSCs) to wounded lung epithelial cells (24). Mitochondrial transcellular transfer by EVs between astrocytes and neurons has also been shown to depend on the NAD+/CD38/cADPR/Ca^2+^ pathway (25, 26). This pathway controls not only the endocytosis stage of EVs, but also seems to be involved in the exocytosis stages, because the amount of EVs is significantly reduced, not increased, after small interfering RNA (siRNA) inhibition of *CD38* expression (25).

The exocyst complex, the critical molecule of actin skeleton remodeling and membrane protrusions formation, as mentioned in section 2.1.2, might also participate in the downstream mechanism of the CD38-mediated EV pathway, because inhibition of the composition of the exocyst complex caused a remarkable reduction in EV formation (70). In addition, the calcium signal that promotes EV formation also participates in TNT formation mediated by the exocyst complex (75). Therefore, we believe that: The exocyst complex can be assembled and activated by the NAD+/CD38/cADPR/Ca^2+^ pathway and interacts with certain types of small GTPase and unknown regulatory factors, leading to the release or endocytosis, or both, of EVs.

### Transfer *via* Gap Junctions

The GJ protein connexin is a widely distributed in various cells. Six rod-shaped connexins form a connexon or hemichannel across the plasma membrane, with a nanoscale hydrophilic channel in the middle. The hemichannel, as a precursor structure of GJs, can also exist independently as a channel connecting the inside and outside of the cell (90, 91). A head-to-head connection between two hemichannels forms a structure called a gap junction channel (GJC) that connects the cytoplasm of two cells. GJCs allow the exchange of ions (especially calcium ions) or small molecules between the two cells (92), facilitating the exchange of chemical or electrical signal between the two cells. GJCs can provide nutrient substances to surrounding cells, protect cells from injury (93), and are involved in clathrin-dependent EV endocytosis (94, 95).

Cx43 is a type of connexin that has been proven to be involved in ischemia/reperfusion injury of myocardial and cerebral tissue, and can also protect myocardial and cerebral tissue from ischemia/reperfusion injury under certain conditions (96). Cx43 seems to have an important association with mitochondrial function. Cx43 hemichannels on mitochondria participate in maintaining mitochondrial calcium homeostasis and can lead to mitochondria damage and cell apoptosis by passing Ca^2+^ (97, 98). Cx43 also participates in the transcellular transfer of mitochondria. In a model of LPS-induced acute lung injury, bone marrow stromal cells (BMSCs) delivered mitochondria to the injured alveolar epithelium. This process relies on Ca^2+^ exchange between the two cells through CX43-GJCs (49).

Cx43 has been proven to be an important regulator involved in the formation and function of TNTs. In a model of airway allergic inflammation, the formation of TNTs connecting induced pluripotent stem cell (IPSC)-MSCs and airway epithelial cells depends on Cx43-GJCs, while downregulating Cx43 expression significantly inhibited the metastatic transfer between the two groups of cells, and simultaneously blocked the protective effect of IPSC-MSCs on lung tissues (12). Cx43 and its related signaling pathways in breast cancer cells, such as Rho associated coiled-coil containing protein kinase (ROCK), protein kinase A (PKA), focal adhesion kinase (FAK) and p38, have novel non-canonical roles of regulating TNT formation (99). Knocking out the CX43 genes of human trabecular meshwork cells also led to a significant reduction in TNTs (100). Cx43-GJCs might play an important role in the formation of TNTs. Cx43-GJCs participate in the coordination mechanism of the two cells. This is likely to be regulated by intercellular ion exchange, especially Ca^2+^, as calcium signal exchange exists between the two cells connected by TNTs (75). Moreover, functional GJs have been found at the end of the membrane extension of TNTs (101), which also supports this hypothesis.

In addition, Cx43-GJCs participate in the intercellular exchange of ROS (102), and there may even be direct mitochondrial transfer *via* Cx43 GJCs (9), which might also be a mechanism for TNT formation and the intercellular transport of mitochondria (**Figure 1C**).

### Transfer *via* Other Routes

TNTs, EVs and Cx43-GJCs are the main routes that mediate transcellular transfer of mitochondria in most studies. However, there are also some other routes, such as mitochondrial extrusion and cytoplasmic fusion.

Naked mitochondria or mitochondrial components can also be extruded and internalized without carrier, which are also called exocytosis and endocytosis (5). Cytoplasmic vacuoles derived from plasma membrane engulf mitochondria and then fuse with cytomembrane to extrude the damaged mitochondria out under the condition of tumor necrosis factor-α (TNFα)-induced apoptosis in a actin and tubulin cytoskeletons-dependent manner (103). Endocytosis of naked mitochondrial was also detected in chloramphenicol (CAP)- and efrapeptin (EF)-sensitive mammalian cells, which internalized free mitochondria isolated from CAP- and EF-resistant fibroblasts in a coincubation medium (104). By endocytosis, vitality and bioenergetics of recipient cells restore (105).

Cytoplasmic fusion is a common phenomenon in which the membrane of two or more cells fuse together and the organelles are shared, in which injury and inflammation may induce this process (106). Cell fusion can regulate the potential of stem cells, which plays an important role in regeneration and oncogenesis (107). Mitochondrial transfer may participate this process (108). However, the extent to which mitochondrial transfer promotes the potential of stem cells is still unknown.

## The Transport Complex Drives Intracellular and Intercellular Movement of Mitochondria

### Movement of Mitochondria Based on Microtubules and Microfilaments

**Figure 4:**
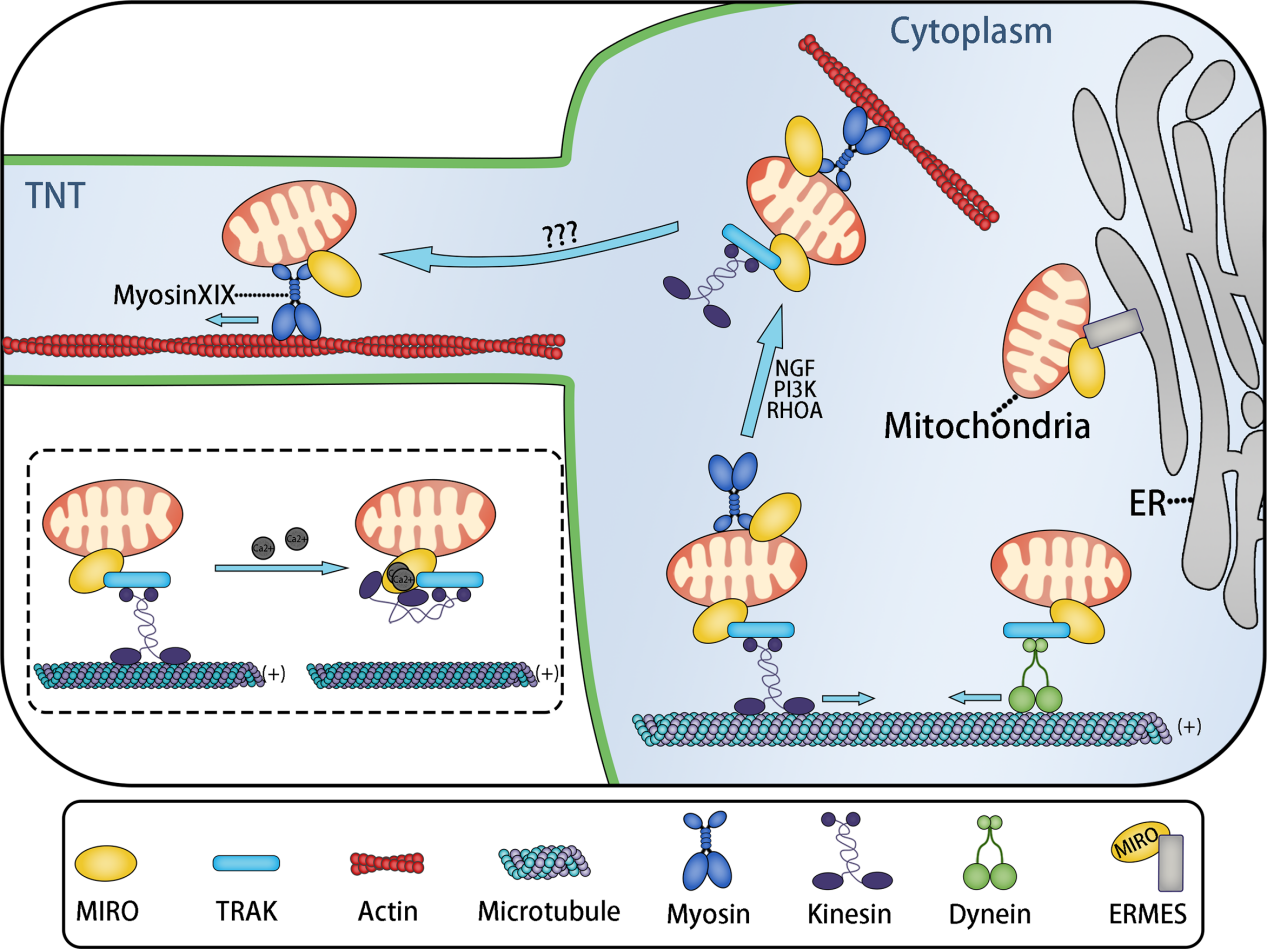
MIRO Mediates the Intracellular and Intercellular Distribution of Mitochondria. Mitochondria move to adapt the ATP demand of different parts of cells. Long-distance transport of mitochondria is usually mediated by microtubules, while short-distance transport of mitochondria is mediated by actin. MIRO might be a key protein involved in mitochondrial transport. MIRO participates in the formation of various mitochondria transport complexes. MIRO/TRAK/kinesin and MIRO/TRAK/dynein transport complexes mediate mitochondrial movement to the plus (+) and minus (−) ends of microtubules, respectively. The ERMES complex, containing MIRO, tethers mitochondria to the endoplasmic reticulum. This might be related to Ca^2+^ exchange. Under the stimulation of nerve growth factor (NGF), PI3K, and RHOA signals, mitochondrial transport changes from microtubule-based transport to microfilament-based transport, completing the actin−based docking of mitochondria. Actin-based docking of mitochondria might represent the transitional stage of mitochondria entering TNTs, while the mitochondrial movement in TNTs is probably mediated by the MIRO/myosin XIX transport complex. These movements are regulated by the local ATP/ADP ratio. However, the transitional mechanism of mitochondria entering TNTs remains unclear. The dotted box: Two calcium ions bind to the EF-hand Ca^2+^-binding domains of MIRO, resulting in the change of kinesin configuration: The microtubule binding site of kinesin binds to MIRO, leading to the separation of mitochondria from the microtubule.

The mitochondrial transport complex consists of mitochondrial Rho small GTPase (MIRO) and motor proteins based on microtubules or microfilaments. In certain cases, adaptors such as TRAK are also needed (**Figure 4**). MIRO, a member of the Ras superfamily, is located on the mitochondrial outer membrane, attached by its C-terminal hydrophobic transmembrane domain (109, 110), and binds with motor proteins directly or indirectly (via adaptor proteins) (43). Transport complex allows mitochondria immobilized on cytoskeleton and provides the power driving the movement of mitochondria.

MIRO1 and MIRO2 are two subtypes of MIRO in humans. The combination of drosophila MIRO (dMIRO) and Milton mediates the transport of mitochondria based on microtubules in Drosophila (111). Inhibition of dMIRO expression leads to mitochondrial transport disorder in the dendrites and axons of Drosophila neurons (112, 113). As the mitochondrial receptor, MIRO forms the transport complex with Milton and Kinesin to mediate the anterograde transport of mitochondria in axons, in which Milton act as a motor adaptor and Kinesin is a type of motor protein based on microtubules (114). Trafficking Kinesin Protein 1 (TRAK1) and TRAK2, the human Milton orthologs, have also been proven to play an important role in the process of mitochondrial transport by binding with MIRO to form MIRO/TRAK/Kinesin complex (115, 116) (**Figure 4**). Indeed, not only kinesin, but also other microtubule motor proteins, such as kinesin superfamily KIF5 and dynein can also interact with TRAK and MIRO to form different mitochondrial transport complexes based on microtubules (43, 117, 118). MIRO contains two GTPase domains (located at the N-terminus and C-terminus, respectively) and two EF-hand Ca2+-binding domains (109, 110). Based on the two EF-hand Ca^2+^-binding domains, Ca^2+^ can change the configuration of the MIRO/adaptor/motor complex to regulate mitochondrial fixation on microtubules. When the cytosolic Ca^2+^ concentration increases, the MIRO/TRAK/motor complex is separated from the microtubule, causing the mitochondria fall off the microtubule (43, 119, 120) (**Figure 4**).

Microtubules are responsible for the long-distance transport of mitochondria, such as the transport in neuron axon, while the short-distance transport mainly depends on the actin microfilament system (43). Mitochondrial transport based on microfilaments is mediated by a transport complex containing actin motor—myosin. Currently known myosin family members that adapt to mitochondria include myosin II, myosin V, myosin VI, and myosin XIX.

Myosin II promotes mitochondrial contraction by inducing deformation of the microfilament skeleton during mitochondrial division (121). Myosin V and VI exert a negative regulatory effect on the microtubule-based axon transport of mitochondria. Downregulating the expression of myosin V and VI can significantly accelerate the speed of mitochondrial transport, which is possibly caused by myosin V and VI mediating the actin-based docking of mitochondria (122). By slowing down the speed of mitochondria and anchoring mitochondria to the cytoskeleton, mitochondrial docking maintains the needed quantity of stationary mitochondria in the regions that need energy and Ca2+-buffering capacity (43). Thus, these regions obtain sufficient ATP. Although the docking receptor has not been identified, it is established that myosin mediates mitochondrial docking (43). This process might also assist the transcellular transport of mitochondria, because the actin-based docking of mitochondria can slow down the speed of mitochondrial movement, which might be a transitional stage in which mitochondria move from the cytoplasm to TNTs or EVs *via* actin microfilaments (**Figure 4**).

### Myosin XIX Drives the Movement of Mitochondria in TNTs

Myosin XIX is a high-duty ratio actin motor with strong affinity for mitochondria (123, 124), which is fixed to the mitochondrial outer membrane *via* a 30–45-residue motif (125), while MIRO can recruit and stabilize myosin XIX to the mitochondrial outer membrane as the mitochondrial motor adaptor protein, thus driving the actin-based movement of mitochondria by interacting with myosin XIX (126, 127). This process is regulated by the local ATP/ADP ratio (128). We hereby propose a hypothesis: The movement of mitochondria in TNTs is driven by the MIRO/myosin XIX transport complex, of which myosin XIX is the motor protein (**Figure 4**).

This hypothesis is based on the following evidence: a. Under the induction of starvation culture or ROS, cells produce abundant filopodia, in which mitochondria exist, and the process of mitochondrial transfer to the top of filopodia has been proved to be mediated by myosin XIX (125, 128, 129); b. As mentioned above, filopodia have many similarities with TNTs: Filopodia and TNTs are both generated by membrane protrusions under oxidative stress and use actin filaments as skeletons. In certain conditions, filopodia can also be converted into TNTs (62); c. Evidence shows that myosin drives TNT-based transcellular material transport (130); and MIRO also mediates TNT-based transcellular mitochondrial transport from mesenchymal stem cells to epithelial cells (21, 131) and nerve cells (132, 133). This process depends on a unique residue (a class specific tryptophan) of the myosin XIX motor domain (129). However, direct evidence of myosin XIX’s presence in TNTs is still needed. Besides, myosin XIX controls actin-based mitochondrial movement during cell mitosis to ensure the symmetrical distribution of mitochondria to daughter cells (134).

### Coordination of MIRO in Transcellular Mitochondrial Transport

MIRO, the connecting component of the transport complex, interacts with microtubules and actin motor proteins such as kinesin and myosin, adaptor proteins TRAK1 and TRAK 2, PTEN induced kinase 1 (PINK1) (regulates mitochondrial autophagy) (135), hypoxia up-regulated mitochondrial movement regulator protein (HUMMR) (involved in mitochondrial movement in neurons under hypoxia) (136), and abundant types of cytoskeleton binding proteins. MIRO can also be used as part of the ER-mitochondria encounter structure (ERMES) complex to fix mitochondria on the endoplasmic reticulum (**Figure 4**) (137, 138). By connecting mitochondria with different transport complexes, MIRO regulates mitochondrial movement based on microtubules and actin, and the transformation between the two forms of mitochondrial movement (126, 127). MIRO not only modulates the intracellular movement of mitochondria, but also mediates the intercellular movement of mitochondria in TNTs.

The evidence above suggests that MIRO is the key protein that modulates intra- and intercellular mitochondria distribution. MIRO might also mediate the transitional stage during which mitochondria move from the cytoplasm to the transcellular transfer structures, such as TNTs or EVs. Mitochondria are separated from the microtubule binding site, and then anchored on an actin filament. After that, mitochondria move to enter TNTs driven by myosin, where mitochondria are anchored on the actin filament of TNTs for subsequent intercellular transport. All these processes are mediated by the transport complexes composed of MIRO and its adaptor proteins and motors (**Figure 4**). Similarly, MIRO might also play an important role in the process of mitochondrial entry into EVs.

## Conclusions

Communication between cells involves the exchange of information (small molecules, ions, complexes, extracellular vesicles, and even various organelles) through various structures such as GJs, TNTs, EVs, endocytosis, and exocytosis. Transcellular transfer of mitochondria is considered a form of intercellular communication that exists commonly in organisms. Cells under stress receive active mitochondria *via* TNTs, EVs, or GJCs to improve their aerobic respiration and viability, which might represent the self-protection capacity of cells. The use of autologous or infused active mitochondria to rescue dying cells seems to be a prospective research direction for the treatment of ischemic/reperfusion-related deficiency. In animal models of stroke, preliminary experiments associated with mitochondrial transcellular transfer have shown some progress (25, 139, 140), with the potential to develop into clinical therapy. This therapy is called mitochondrial transplantation, which has shown promising effects in several central nervous system (CNS) diseases, such as cerebral ischemia and Parkinson’s disease (4). Moreover, tumor cells can also convert their metabolism to enhance proliferation or apoptosis *via* the transcellular uptake of mitochondria, which might also be a potential therapeutic target of anti-tumor drugs. Intervention could be possible at multiple nodes in these processes: The formation of TNTs or EVs; the receipt of TNTs and EVs by the target cell; the function of Cx43-GJs; and the transport complex driving mitochondrial movement.

Thee NAD+/CD38/cADPR/Ca^2+^ pathway and the exocyst complex might participate in regulating the formation of TNTs and EVs; however, currently, the specific regulation mechanism is poorly understood. In particular, the detailed mechanisms of the fusion of TNTs and the target cell membrane, the receipt of signals by surrounding cells signals and the release of EVs, the endocytosis of EVs by target cells, the role of GJs and the cytoskeleton in TNTs and EVs, the accurate regulation of mitochondrial transfer based on microtubules and microfilaments, and the entry of mitochondria remain to be determined in future studies.

## Author Contributions

YQ wrote the manuscript and drew the figures. XJ and QY collected the related papers, created the Tables, and helped to revise the manuscript. XJ, QY, and JZ participated in the design of the review. YZ and QZ designed and revised the manuscript. All authors contributed to the article and approved the submitted version.

## Funding

This work was supported the Science and Technology Foundation Survey Project of Ministry of Science and Technology of China [grant numbers 2018FY100900 and 2018FY10090004].

## Conflict of Interest

The authors declare that the research was conducted in the absence of any commercial or financial relationships that could be construed as a potential conflict of interest.

## Abbreviations

TNTs, tunneling nanotubes; EVs, extracellular vesicles; GJs, gap junctions; GJCs, gap junction channels; MSCs, mesenchymal stem cells; ROS, reactive oxygen species; OXPHOS, oxidative phosphorylation; DNA, deoxyribonucleic acid; ATP, adenosine triphosphate; BMSCs, bone marrow stromal cells; CMs, cardiomyocytes; hMADs, human multipotent adipose-derived stem cells; RTCs, renal tubular cells; MMSCs, mesenchymal multipotent stromal cells; hVSMCs, human vascular smooth muscle cells; MELAS, mitochondrial myopathy, encephalomyopathy, lactic acidosis, and stroke-like episodes; LST1, leukocyte specific transcript 1; RalBP1, Ral binding protein 1; GEF, guanine nucleotide exchange factor; MDRCs, myeloid-derived regulatory cells; cADPR, cyclic ADP-ribose; NAD, nicotinamide adenine dinucleotide; RyRs, Ryanodine receptors; ROCK, Rho associated coiled-coil containing protein kinase; FAK, focal adhesion kinase; TNFα, tumor necrosis factor-α; MIRO, mitochondrial Rho small GTPase; TRAK, Trafficking kinesin protein; KIF5, Kinesin family member 5; ERMES, ER-mitochondria encounter structure; HUMMR, hypoxia up-regulated mitochondrial movement regulator; NGF, nerve growth factor.
